# Mammographic Breast Density of Japanese Women Living in Australia: Implications for Breast Screening Policy 

**DOI:** 10.31557/APJCP.2019.20.9.2811

**Published:** 2019

**Authors:** Miwa M Mizukoshi, Syeda Z Hossain, Ann Poulos

**Affiliations:** 1 *Discipline of Behavioural and Social Sciences in Health,*; 2 *Discipline of Medical Radiation Sciences, The University of Sydney, Australia. *

**Keywords:** Breast density, mammography, Japan, Australia, early detection of cancer

## Abstract

**Background:**

Mammographic Breast Density (MBD) increases breast cancer risk, lowers sensitivity of mammography and is related to ethnicity. This study compared the MBD of Australian women living in Australia (AW), Japanese women living in Japan (JWJ) and Japan-born women living in Australia (JWA). The outcomes have implications for breast screening policies in Australia.

**Methods:**

In this cross-sectional study, mammographic images were collected from a total of 677 women who were examined at BreastScreen NSW or Miyata Hospital, Japan. The images were retrospectively evaluated using the 5^th^ edition Breast Imaging-Reporting and Data System (BI-RADS) to assess the level of MBD. Descriptive analyses and binary logistic regressions were performed.

**Results:**

More than 65% of AW had low MBD while the majority of JWJ had high MBD. Among AW, low MBD was found in women aged 40-49 and 50-59. The MBD of JWA showed a similar pattern to JWJ but with a slightly lower MBD. The great majority of JWA aged 40-49 were found to have high MBD compared to women aged 50-59. JWJ were categorised as high MBD regardless of their age. JWA were 5 times more likely and JWJ were 15 times more likely to have high MBD compared to AW.

**Conclusion:**

Mammography screening becomes more effective for JW after migration to Australia. For JWA aged 40-49 years, supplemental screening options are indicated to provide better cancer detection. For AW, screening mammography could be equally as effective for AW aged 40-49 years as for those aged 50-59.

## Introduction

Since breast cancer is the most commonly diagnosed female cancer in Australia and Japan, population-based breast cancer screening is conducted nationally in both countries (Australian Institute of Health and Welfare, 2012; National cancer centre Japan, 2014). Comparison of breast cancer incidence (BCI) rates in Australia and Japan shows that the BCI peaks in Australia for women aged 65-69 years and in Japan for women aged 45-49 (Matsuda et al., 2010; Albeshan et al., 2018b). Therefore, Breastscreen Australia targets women aged 50-74 for screening mammography and the Japanese Breast Cancer Society targets women aged 40-74 (Japanese Breast Cancer Society, 2015; Australian Government, 2016). Previous BCI studies in multi-ethnic countries have reported that BCI rates vary between ethnicities, and BCI rates among migrants did not exhibit the same characteristics of either their country of origin or their host country (Maskarinec and Noh, 2004; Albeshan et al., 2018b). Women of Asian descent living in North America have a markedly increased BCI rate (Health Quality Ontario, 2007), and the BCI rates for Japanese American women have also demonstrated a rapid increase. In addition, Japanese women who have migrated to the United States (US) have a higher BCI rate than Japanese women living in Japan (Deapen et al., 2002).

Mammographic breast density (MBD) is an important predictor of breast cancer risk and affects the diagnostic performance of mammography. MBD is the amount of white radiopaque area, which consists of fibroglandular tissue, relative to the darker radiolucent area, consisting of adipose or fatty tissue, on a mammogram. High amounts of MBD compromise the diagnostic accuracy of mammograms and can be responsible for false-positive and false-negative results (Boyd et al., 2011). MBD can be measured qualitatively by Wolfe, Boyd, Tabár and BI-RADS (Breast Imaging Reporting and Data System) methods which are significant visual classification methods. Quantitative methods that provide objective outcomes include computer-assisted volumetric breast density measurement using software such as Volpara Density and Quantra (Highnam et al., 2010; Nickson et al., 2012).

High MBD is predominantly found in younger women and typically decreases with age (Checka et al., 2012). This is not always the case, however, and breast density in some women remains high after menopause (Checka et al., 2012). Thus, the relationship between age and MBD is not fully understood. Among women aged 40-49 years with high MBD in the US, harms from screening mammography were highest in women with high MBD and lowest in women with lower MBD (Nelson et al., 2016). Currently, Australian guidelines for screening mammography state that Australian women aged 40-49 years are not actively recruited due to their increased volume of fibroglandular tissue (Australian Government Department of Health, 2013).

Racial/ethnic differences in MBD have previously been reported in multiple studies. Quantitative MBD assessment in the US showed black women had higher MBD than white women (McCarthy et al., 2016). Other US studies showed women with Asian background had significantly higher MBD than Caucasian/White women, while lowest MBD was seen in African-American women (El-Bastawissi et al., 2001; Ursin et al., 2003; del Carmen et al., 2007). Lifestyle has also been found to be associated with MBD. Individual variables such as body mass index (BMI), hormone replacement therapy (HRT), menopausal status, family history, tamoxifen intake, parity, diet and use of oral contraceptives are known from earlier studies (Byrne, 2002; Pape et al., 2017). An increase in BMI, tamoxifen therapy and parity decreases breast density (Freer, 2015) whereas use of HRT increases breast density (Freer, 2015). 

Migrant women adopt different lifestyles in their new country and their MBD may show unique characteristics. MBD of migrant Ethiopian women in Israel was reported to vary according to length of residency (Sklair-Levy et al., 2017). Migrant Hispanic women had lower MBD than US-born women, (Tehranifar et al., 2018) and migrant Chinese women in the US with most acculturation had high MBD (Tseng et al., 2006). Women living in Australia who were born in countries with predominantly Asian ancestry had high MBD (Bell et al., 2019). In Japan, high MBD was observed in 78% of non-symptomatic women and 87% of breast cancer patients using a quantitative MBD measurement (Sawada, 2017). A study comparing the MBD of Japanese women in Hawaii and Japan reported that the former had significantly higher percent densities than the latter (Maskarinec et al., 2002). 

The aim of this study was to compare the MBD of Australian women living in Australia (AW), Japanese women living in Japan (JWJ) and Japan-born women living in Australia (JWA). Information about variations among the three ethnic groups can inform policy makers on the effectiveness of mammographic screening in different ethnicities due to the masking effect. The outcomes of the study also suggest implications for current screening policies for migrant women who make lifestyle changes in their new country. 

## Materials and Methods

Ethical approval was obtained from the NSW (New South Wales) Population and Health Services Research Ethics Committee (AU RED Reference: HREC/16/CIPHS/26). Access to the data was approved by Breast Screen NSW (Australia) and Miyata Hospital (Japan).

*Sample*

This cross-sectional study retrospectively assessed images from a total of 677 women. In this study, AW are defined as women who speak English at home and were born in Australia, JWA are those who speak Japanese at home, were born in Japan but were now living in Australia and had participated in mammographic screening, and JWJ are those who were born and living in Japan who had participated in mammographic screening at Miyata Hospital in Japan. The participants’ ages ranged from 40-69 years and were placed into 5-year age groups.

Right and left cranio-caudal (CC) and medio-lateral oblique (MLO) mammography images taken for the purpose of breast cancer screening between 2014 and 2015 were used for the MBD analysis. Self-reported demographic data were collected for each participant. All data were de-identified by the Cancer Institute NSW before readers received them for data analysis. 

Exclusion criteria were set for mammograms that showed abnormal lesions or were inadequately positioned, since these images can affect the accuracy of breast density classification. Also excluded were sets of the images of the same women who attended screening more than once between 2014 and 2015, women with breast augmentation, including implants, and women with a history of surgery, including lumpectomy, mastectomy, reconstruction and breast reduction. 

**Figure 1:**
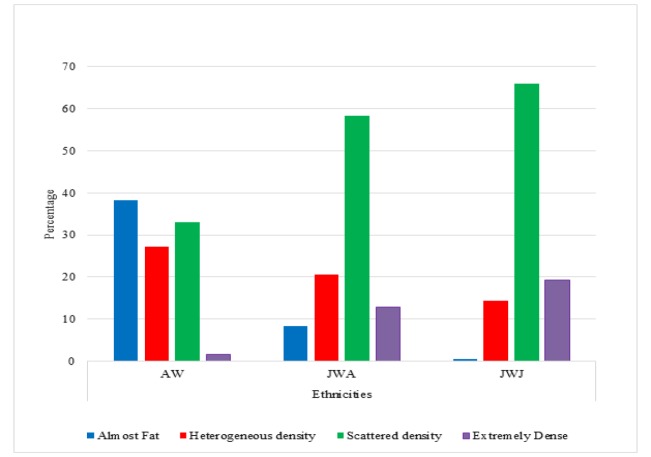
MBD of Ethnic Groups and Place of Residence by BI-RADS Category

**Table 1:** Characteristics of Study Population by Ethnicity

Characteristics	Australian women N (%)	Japanese women in Australia N (%)	Japanese women in Japan N (%)
Participation	203 (30)	202 (29.8)	272 (40.2)
Age			
40-44	38 (19)	45 (22.3)	77 (28.3)
45-49	62 (31)	58 (28.7)	49 (18)
50-54	26 (13)	37 (18.3)	50 (18.4)
55-59	26 (13)	26 (12.9)	55 (20.2)
60-64	24 (12)	23 (11.4)	33 (12.1)
65-69	24 (12)	13 (6.4)	8 (2.9)
Total	200 (100)	202 (100)	272 (100)
HRT ª			
Yes	20 (9.9)	9 (4.5)	
No	182 (90.1)	193 (95.5)	
Total	202 (100)	202 (100)	
Significant family history ᵇ			
Yes	19 (9.5)	14 (7.1)	1 (0.5)
No	181(90.5)	184 (92.9)	207 (99.5)
Total	200 (100)	198 (100)	208 (100)
History of breast cancer			
Yes	5 (2.5)	1 (0.5)	3 (1.1)
No	192 (97.5)	192 (99.5)	269 (98.9)
Total	197 (100)	193 (100)	272 (100)
Self-reported symptoms			
Yes	12 (6.1)	15 (7.7)	18 (6.6)
No	186 (93.9)	179 (92.3)	253 (93.4)
Total	198 (100)	194 (100)	271 (100)
Screening result			
Negative	190 (95)	182 (91.9)	237 (87.5)
Recall for assessment	10 (5)	16 (8.1)	34 (12.5)
Total	200 (100)	198 (100)	271 (100)

ª Hormone Replacement Therapy, ᵇ First degree relatives with breast cancer

**Table 2:** Distribution of MBD by Age and Ethnic Group

Age group	Ethnic group	BI-RADS Category N (%)		Total N(%)
		*a or b	**c or d	
40-49	Australian women	59 (61.5)	37 (38.5)	96 (100)
	Japanese women living in Australia	20 (19.8)	81 (80.2)	101 (100)
	Japanese women living in Japan	14 (11.1)	112 (88.9)	126 (100)
	Total	93 (28.8)	230 (71.2)	323 (100)
50-59	Australian women	32 (66.7)	16 (33.3)	48 (100)
	Japanese women living in Australia	21 (35)	39 (65)	60 (100)
	Japanese women living in Japan	10 (9.5)	95 (90.5)	105 (100)
	Total	63 (29.6)	150 (70.4)	213 (100)
60-69	Australian women	34 (73.9)	12 (26.1)	46 (100)
	Japanese women living in Australia	15 (44.1)	19 (55.9)	34 (100)
	Japanese women living in Japan	7 (17.1)	34 (82.9)	41 (100)
	Total	56 (46.3)	65 (53.7)	121 (100)

*a or b, less dense breasts and mammography is more sensitive; **c or d, dense breasts and mammography is less sensitive.

**Table 3:** Odds Ratios (ORs)ᵃ using Dichotomous MBD Classificationᵇ by Ethnic Group and Age

		Unadjusted OR (95% CI)ᶜ	Statistical Significance (p)	Adjusted OR (95% CI)	Statistical Significance (p)
Ethnic group	Australian	1.0 (ref.^d^)		1.0	
	Japanese in AUS	4.8 (3.1-7.4)	<0.0005	4.9 (3.1-7.5)	<0.0005
	Japanese in JPN	15.1 (9.3-24.3)	<0.0005	15.8 (9.7-25.7)	<0.0005
Age	≥50	1.0 (ref.^d^)		1.0	
	40-49	1.4 (1.0-1.9)	0.06	1.7 (1.1-2.4)	0.01

a. OR, Odds Ratio of high MBD (b-c category) relative to less MBD (a-b category); b. Two-category scale classification includes “almost entirely fatty or scattered ﬁbroglandular breast tissue” or “heterogeneously or extremely dense breast”; c. 95% CI, 95% conﬁdence interval; d. Reference.

*Data collection*

Breast density was assessed qualitatively according to the Breast Imaging-Reporting and Data System (BI-RADS) mammographic breast composition categories 5th edition, published by the American College of Radiology (ACR) (Sickles EA, 2013). The 5th edition uses a subjective method that does not provide percentage MBD in order to emphasise information about the likelihood of the lesion (s) being obscured by fibroglandular tissue (Sickles EA, 2013; Irshad et al., 2016).

The images were categorised into four groups: (a) The breasts are almost entirely fatty; (b) There are scattered areas of fibroglandular density; (c) The breasts are heterogeneously dense, which may obscure small masses; and (d) The breasts are extremely dense, which lowers the sensitivity of mammography (Sickles EA, 2013). Three specialist mammography clinicians with 10-20 years’ experience completed the categorisation independently. Reader 1 was a specialist mammography radiographer with more than 10 years’ experience who practised in Australia and Japan, Reader 2 was a breast imaging specialist radiologist in Japan, and Reader 3 was a specialist mammography radiographer with more than 20 years’ experience in Australia. All cases (n=677) were blindly assessed by Reader 1, the cases from Japan (n=272) were classified by Reader 2, and cases from Australia (n=405) were rated by Reader 3. A single MBD score of each case was identified by taking the highest score from all available views, as recommended in the ACR BI-RADS Atlas (Sickles EA, 2013).

*Data analysis*

Inter-reader agreement among the three readers was tested using Weighted Kappa for the assessment in four- (a, b, c or d) and two- (a-b or c-d) BI-RADS density category scales, as described by Ekpo et al., (2016) (Ekpo et al., 2016). Kappa values were interpreted by the Landis and Koch criteria, in which values < 0.00 are categorised as ‘Poor’, 0.00-0.20 as ‘Slight’, 0.21-0.40 as ‘Fair’, 0.41-0.60 as ‘Moderate’, 0.61-0.80 as ‘Substantial’, and 0.81-1.00 as ‘Almost Perfect’ agreement.

Descriptive analysis was carried out for the distribution of the background characteristics of the study population. Associations between MBD distribution, ethnicity and age were tested using Kruskal-Wallis and chi-square tests for statistical significance, and Spearman’s rho correlation for the strength of the association. Binary logistic regression was performed to compare odds ratios (OR) stratified by ethnicity (AW/JWA, AW/JWJ) and age group (≥50 / <50). Multicollinearity testing was used to assess inter-associations among the independent variables. IBM® SPSS® Statistics (version 24) was used for data analysis.

## Results

*Inter-reader agreement*

Inter-reader agreement between Reader 1 and Reader 2 showed weighted kappa value was 0.77 and fell into the “substantial” range (95% CI, 0.70-0.84) using the four-category density scale. Weighted kappa value on a two-category density scale was 0.83 (95% CI, 0.73-0.93) and indicating the “almost perfect”. Weighted kappa values between Reader 1 and Reader 3 showed 0.77 (95% CI, 0.70-0.83) on a four-category scale and 0.84 (95% CI, 0.75-0.92) on a two-category scale which were similar values as those for Reader 1 and 2. Therefore, the MBD score by reader 1 was used for this analysis.

*Background characteristics*

Of the 677 women, three either did not have age information or had duplicated information and were excluded from this study (Table 1). The proportion of Japanese women living in Japan was slightly higher, but the distribution of participants in each ethnic group was similar. Women aged under 50 comprised 50% of the total participants, and this was similar across the ethnic groups. Data on HRT status among Japanese women living in Japan were not available due to the retrospective nature of the study. The proportion of women on HRT was, however, very small. Other self-reported information, such as significant family history of breast cancer, history of breast cancer, symptoms and recall for assessment, was available but the sample size was also very small. There was no multicollinearity problem between the variables (tolerance > 0.1, variance inflation factor < 10), indicating that there was no inter-association among variables.

*Ethnicity and MBD*

The distribution of MBD using the four-category BI-RADS scale among the three groups (AW, JWA and JWJ) is shown in Figure 1. Approximately 40% of AW were in the “almost entirely fatty breast” category, while 66.5% and 22% of the JWJ were in the “heterogeneously dense” and “extremely dense” categories, respectively. JWA showed a similar distribution pattern to those living in Japan but slightly fewer women were in dense categories, with 58.5% “heterogeneously dense” and 12.8% “extremely dense”. A Kruskal-Wallis test showed that the differences in MBD among the ethnic groups were statistically significant (AW p<0.001, JWA p<0.001 and JWJ p<0.001).

*Age and MBD*

Table 2 shows the distribution of MBD by age group using BI-RADS two-scale categories. In two-scale categories, extremely and heterogeneously dense breasts are considered high MBD and breasts with almost entirely fat and scattered fibroglandular densities are categorised as low MBD (Ekpo et al., 2016). More than 80% of JWJ were in the high MBD category regardless of their age (40-49: 88.9%, 50-59: 90.5%, 60-69: 82.9%). A relatively high percentage of JWA aged 40-49 also had high MBD (80.2%), compared to women aged 50-59 (65%) and 60-69 (55.9%). In contrast, the majority of AW were in the low MBD category across all age groups (40-49: 61.5%, 50-59: 66.7%, 60-69: 73.9%).

Spearman’s Rho test showed a weak negative correlation between age and MBD in each ethnic group, with correlation coefficient -0.211 (p=0.004) for AW, -0.265 (p<0.001) for JWA and -0.202 (p<0.001) for JWJ. This indicates that MBD decreased with age in all three groups. In addition, chi-square found that ethnicity was significantly associated with BI-RADS MBD within each age group (40-49: χ^2^= 73.158, p<0.001, χ^2^= 50-59: 52.82, p<0.001 and 60-69: χ^2^= 28.26 p<0.001).

*Odds ratios*

Unadjusted OR of having higher MBD in JWA compared with AW was 4.812 (95% CI 3.1-7.4, P<0.0005), as shown in Table 3. This indicates that JWA were 4.8 times more likely to have high MBD compared with AW (reference). Unadjusted OR in JWJ was 15.1 (95% CI, 9.3-24.3), indicating that JWJ were 15 times more likely to have high MBD compared with AW (P<0.0005). After adjusting for age, the OR of JWA and JWJ did not show a substantial change, with 4.9 (95% CI, 3.1-7.5) and 15.8 (95% CI, 9.7-25.7), respectively. 

Although unadjusted OR of women aged 40-49 years was 1.4 when women aged over 50 years were set as a reference group, the confidence interval included 1.0 (95% CI 1.0-1.9). This indicates that the higher MBD seen in women younger than 50 was equally probable for women aged 50 and older. The result was also marginally significant (p=0.06). After controlling ethnicity, the adjusted OR for women aged 40-49 was 1.7 and was statistically significant (95% CI 1.1-2.4, p=0.01).

*HRT and MBD*

Of the 386 women who self-reported HRT status, 191 were AW and 195 were JWA, and only 10% of AW (n=19) and 4% of JWA (n=8) were current and past HRT users. More than 70% of HRT users among AW (n=14) had low MBD, whereas more than 60% of HRT users among JWA (n=5) had high MBD. Cramer’s V test showed that there was a weak but positive association between the use of HRT and MBD (Cramer’s V: AW =0.054, JWA=0.04). This indicates that MBD increased when women used HRT. Because of the small sample size, Fisher’s exact test was also conducted. The correlation was, however, not statistically significant (AW=0.612, JWA p=0.692). 

## Discussion

The study showed AW were more likely to have low MBD (65%), whereas high MBD was seen in the majority of JWJ (90%). The high MBD result among JWJ was consistent with findings by Sawada (2017) (Sawada, 2017). Odds ratios provided further evidence of differing MBD between ethnicities. When MBD of JWA and JWJ were compared to AW, unadjusted ORs showed JWA were 4.8 times more likely and JWJ 15 times more likely to have high MBD compared to AW. A previous study comparing the MBD of JWJ, JW living in Hawaii and Caucasian women in Hawaii showed that the percent density was highest in JWJ, followed by JW in Hawaii and was lowest in Caucasian women (Maskarinec et al., 2002). Another study using BI-RADS 5th edition for MBD measurement showed that more Asian women in the United Arab Emirates were classified in the higher MBD category than Western women (Albeshan et al., 2018a). 

A previous study reported that breast size differed by ethnicity and size of fibroglandular area changed according to place of residence (Maskarinec et al., 2002). This study observed a similar pattern of MBD distribution in JWA and JWJ, but JWA had slightly lower MBD compared to JWJ. Although breast size was not measured in the present study, if breast size is assumed to be the same between JWA and JWJ, the size of the fibroglandular area was smaller for Japanese women residing in Australia than for those living in Japan. This implies that screening could be more effective for JWA than JWJ as MBD could decrease after migration to Australia.

The reduced MBD of JWA is possibly caused by changes in diet, BMI, and use of HRT and oral contraceptives in their new country, all of which are associated with MBD (Byrne, 2002; Freer, 2015; Pape et al., 2017). A previous study indicated that lifestyle changes among JWA could result from their migration experiences. JWA are not only business migrants but also cultural migrants who seek new ways of life, as well as marriage migrants, who live in their partners’ home country. Therefore, they are actively engaged in local communities and attempt to redefine their lives (Hamano, 2014). 

Under current screening practice in Australia, JWA receive mammography screening as per BreastScreen Australia’s recommendations, regardless of their MBD profile. Supplemental screening can be considered to improve cancer detection. A randomised controlled trial in Japan found that ultrasonography adjunct screening provided higher sensitivity of cancer detection and improved screening effectiveness (Ohuchi et al., 2016). Digital breast tomosynthesis (DBT) is an emerging screening technology that reduces recall rates and increases cancer detection rates (Skaane, 2017). Thus, screening practice recommendations should take ethnicity into account.

The inverse relationship between age and MBD seen in this study was consistent with previous knowledge. (Checka et al., 2012). Ethnicity-adjusted ORs showed women 40-49 are 1.7 times more likely to have high MBD than women 50 and older. Additionally, each ethnic group showed slightly different MBD distributions by women’s age. An overwhelmingly high proportion of JWJ across all age groups in this study demonstrated high MBD. Among JWA, more than 80% of women aged 40-49 years also had high MBD, followed by those aged 50-59 and 60-69 years. In contrast, 62% of AW aged 40-49 years were classified as low MBD, which was similar to those aged 50-59 (67%). A previous MBD study in the UAE suggested that screening could be effective for Emirati women due to their low MBD (Albeshan et al., 2018a). Our results also suggest that screening is equally as effective for AW aged 40-49 as for those aged 50+ due to their low MBDs. 

The descriptive analysis showed that HRT was associated with high breast density, which was consistent with prior knowledge (Boyd et al., 2011). However, the relationship was weak and statistically non-significant. This could be due to the small sample size of HRT users and limited data on the other confounders. An earlier study reported that, among women who were overweight or obese, HRT use was not associated with high MBD (Couto, 2012; Hou et al., 2013). Further HRT and MBD studies focusing on interactions among other factors may provide new insights into the role of HRT in a selected population.

There are strengths and limitations in this study. The availability of data from Australia and Japan provided a novel opportunity to investigate MBDs of two different ethnicities in different residential locations. Substantial inter-reader agreement was found using the four-category scale and almost perfect agreement using two category scales, which enabled the MBD assessment to be conducted by a single reader. This study also had limitations. First, sample sizes for some risk factors were too small for analysis. Second, not all the relevant confounders were available, such as HRT for JWJ, and BMI and socioeconomic status (education, occupation and income). 

Overall, the results of this study indicate that consideration of women’s ethnic background is important for an effective screening program in Australia, as MBD varied between ethnicities. Although Japanese comprise only one of the many ethnic minorities in Australia, the outcomes of this study provide new insight into the implications of current screening policies for immigrant women from Asian backgrounds who adopt the Australian lifestyle. There are three important implications to be drawn from this study. First, mammography screening becomes more effective for JW after migration to Australia. Second, supplemental screening options, such as ultrasonography and tomosynthesis adjunct screening, are indicated to provide better cancer detection for JWA aged 40-49 years. Third, screening mammography could be equally as effective for AW aged 40-49 years as for those aged 50-59. Further robust research is warranted to develop a comprehensive method of mammographic breast density measurement that considers the risk of the masking effect, to inform recommendations to women following the provision of mammographic breast density information, and to specify the harms and benefits of supplemental screening for women with high breast density.

*Ethical approval*

This study was approved by the NSW Population and Health Services Research Ethics Committee (HREC/16/CIPHS/26). All participants signed consent before undertaking their mammograms at BreastScreen NSW. This research will involve no more than low risk to the participants and privacy is protected by using deidentified images.
